# Functional expression of the ATP-gated P2X7 receptor in human iPSC-derived astrocytes

**DOI:** 10.1007/s11302-023-09957-8

**Published:** 2023-07-15

**Authors:** Jaideep Kesavan, Orla Watters, Laura de Diego-Garcia, Aida Menéndez Méndez, Mariana Alves, Klaus Dinkel, Michael Hamacher, Jochen H. M. Prehn, David C. Henshall, Tobias Engel

**Affiliations:** 1 Department of Physiology & Medical Physics, RCSI University of Medicine & Health Sciences, Dublin, D02 YN77 Ireland; 2 FutureNeuro, SFI Research Centre for Chronic and Rare Neurological Diseases, RCSI University of Medicine and Health Sciences, Dublin, D02 YN77 Ireland; 3 Department of Science & Computing, SETU Waterford, Cork Rd., Co., Waterford, X91 K0EK Ireland; 4 Department of Optic and Optometry, Faculty of Optics and Optometry, Complutense University of Madrid, Madrid, 28037 Spain; 5 Lead Discovery Center GmbH, Otto-Hahn-Straße 15, 44227 Dortmund, Germany; 6 Affectis Pharmaceuticals AG, Otto-Hahn-Straße 15, 44227 Dortmund, Germany

**Keywords:** P2X7 receptor, hiPSC-derived astrocytes, ATP and BzATP-evoked Ca^2+^ fluctuations

## Abstract

Activation of the ATP-gated P2X7 receptor (P2X7R), implicated in numerous diseases of the brain, can trigger diverse responses such as the release of pro-inflammatory cytokines, modulation of neurotransmission, cell proliferation or cell death. However, despite the known species-specific differences in its pharmacological properties, to date, most functional studies on P2X7R responses have been analyzed in cells from rodents or immortalised cell lines. To assess the endogenous and functional expression of P2X7Rs in human astrocytes, we differentiated human-induced pluripotent stem cells (hiPSCs) into GFAP and S100 β-expressing astrocytes. Immunostaining revealed prominent punctate P2X7R staining. P2X7R protein expression was also confirmed by Western blot. Importantly, stimulation with the potent non-selective P2X7R agonist 2′,3′-O-(benzoyl-4-benzoyl)-adenosine 5′- triphosphate (BzATP) or endogenous agonist ATP induced robust calcium rises in hiPSC-derived astrocytes which were blocked by the selective P2X7R antagonists AFC-5128 or JNJ-47965567. Our findings provide evidence for the functional expression of P2X7Rs in hiPSC-derived astrocytes and support their in vitro utility in investigating the role of the P2X7R and drug screening in disorders of the central nervous system (CNS).

## Introduction

The P2X7 receptor (P2X7R) belongs to the cationic P2X receptor family and plays a pivotal role in ATP-mediated signal transmission in the brain [1]. The downstream consequences of P2X7R signaling are dependent on its cellular location and various non-cell autonomous effects have been reported. P2X7R activation on microglia leads to the release of pro-inflammatory mediators (e.g. interleukin-1β (IL-1β), IL-6, tumor necrosis factor-α (TNF-α)), triggering neuroinflammation [2]. In contrast, the presence of P2X7Rs on other brain cell types such as neurons and astrocytes is less certain. Activation of P2X7Rs on astrocytes has been reported to evoke glutamate release leading to potential excitotoxic effects [3]. Astrocyte-mediated P2X7R responses may, however, also be important for the release of pro-inflammatory cytokines [4]. However, other studies have failed to detect P2X7Rs on this cell type [5, 6].

Due to its distinct characteristics, including its relatively low affinity for ATP and its prominent role in driving inflammatory processes, P2X7R has attracted considerable interest as a potential target for various neurological conditions including neurodegenerative and psychiatric diseases, and epilepsy. P2X7R protein expression has been found to be up-regulated in the brain of patients with different brain diseases and P2X7R antagonism has shown benefits in multiple animal models (e.g. epilepsy, schizophrenia, depression) [7].

Despite the 80% sequence homology between human and murine P2X7Rs, differences in receptor sensitivity towards various ligands between humans and rodents have been described. This includes the non-selective agonist 2′, 3′-O-(benzoyl-4-benzoyl)-adenosine 5′- triphosphate (BzATP) [8], the potent P2X7Rs antagonist KN-62 (1‐[N,O‐bis(5‐isoquinolinesulphonyl)‐N‐methyl‐L‐tyrosyl]‐4‐phenylpiperazine) [9], and GW791343, a negative modulator at human P2X7Rs, but a positive modulator at rat P2X7Rs [10]. Thus, P2X7Rs exhibit remarkable species-specific differences in the pharmacological properties, demonstrating the need for the investigation of P2X7Rs in human brain-relevant models. Resected human primary brain tissue is very scarce and often shows alterations associated with pathology. Induced pluripotent stem cell (iPSC)-derived human neurons and glia have demonstrated great potential for the investigation of human neuronal development in vitro, pharmacological screening and assessing phenotypic alterations in patient-specific iPSC-derived neural cells [11].

In rodent brain, although P2X7Rs are preferentially localized on microglia, they are also expressed on other cell types (e.g. oligodendrocytes and most likely astrocytes and neurons). Importantly, P2X7R-mediated effects via astrocytes have been suggested to contribute to disease progression in several brain diseases [12]. To date, only few studies have, however, addressed the functional expression of the P2X7R in human primary astrocytes [13, 14]. Here, we demonstrate the expression of P2X7Rs in hiPSC-derived astrocytes, thereby establishing a model for the investigation of functional P2X7Rs in a human system.

## Methods

### Culture and differentiation of hiPSCs

hiPSC line HPSI0114i-eipl_1 (ECACC 77,650,081; Culture Collections, Public Health England, UK) was maintained under feeder-free conditions on vitronectin (STEMCELL Technologies, British Columbia, Canada)-coated 6-well plates in E8 medium (Thermo Fisher Scientific, Massachusetts, U.S.A). hiPSCs were dissociated by using 0.5 mM EDTA for 2 min at 37 °C, and reseeded at the density of 1 × 10^4^ cells/cm^2^. For neural induction of hiPSCs, approximately 24 h after splitting, culture medium was switched to Gibco PSC Neural Induction Medium (Thermo Fisher Scientific, Massachusetts, U.S.A). At day 10 of neural induction, primitive NSCs (pNSCs) were dissociated with Accutase (Thermo Fisher Scientific, Massachusetts, U.S.A) and plated on Geltrex-coated dishes at a density of 1 × 10^5^ cells/cm^2^ in NSC expansion medium containing 50% Neurobasal medium, 50% Advanced DMEM/F12, and 1% neural induction supplement (Thermo Fisher Scientific, Massachusetts, U.S.A). pNSCs were passaged on the 4th day at a 1:3 split ratio to derive NSCs and cells at passage 4 were used for the differentiation of glia. For astrocyte differentiation, pNSCs were plated onto Geltrex-coated coverslips at a density of 5 × 10^4^ cells/cm^2^ in an astrocyte differentiation medium (DMEM supplemented with 1% fetal bovine serum, 1% sodium pyruvate, 1% non-essential amino acids, 0.5% G-5 supplement (all from Thermo Fisher Scientific, Massachusetts, U.S.A)) for 5 days. Astrocytes were passaged at a split ratio of 1:2 and used for experimentation at passage 7–10.

### Immunocytochemistry

Astrocytes cultured on coverslips were fixed with 4% paraformaldehyde or a combination of acetic acid (6.71%) and ethanol (62.5%) for 15 min. After 3 washes in phosphate buffered saline, cells were permeabilised with 0.1% Triton for 20 min and blocked in 1% BSA for 30 min. Cells were incubated at 4 °C overnight in primary antibody diluted in 1% BSA. Cells were then incubated in secondary antibody diluted in 1% BSA for 1 h at room temperature. Primary antibodies: mouse anti-GFAP (Merck, Missouri, United States, Cat. Nr. G6171, dilution 1:200), mouse anti-S100β (Abcam, Cambridge, UK, Cat. Nr. Ab52642, dilution 1:200), rabbit anti-P2X7R (Alomone Labs, Jerusalem, Israel, Cat. Nr. APR-004, dilution 1:200). The respective secondary antibodies were conjugated to Alexa Fluor 488 or 594 (Thermo Fisher Scientific, Massachusetts, U.S.A). Nuclei were counterstained with Hoechst (Merck, Missouri, United States).

### Drug application

Stock solutions of BzATP (Alomone Labs, Jerusalem, Israel), ATP (Merck, Missouri, United States), AFC-5128 (provided by Affectis Pharmaceuticals AG, Dortmund, Germany) [15] and JNJ-47,965,567 (Alomone Labs, Jerusalem, Israel) were diluted in HEPES-buffered extracellular solution. Drug solutions were delivered to recorded cells by a valve-controlled fast multibarrel superfusion system with a common outlet ~ 350 μm in diameter (Automate Scientific, California, U.S.A) with a final concentration: BzATP, ATP (300 µM), AFC-5128 (30 nM), JNJ-47965567 (100 nM).

### Calcium imaging

Cells were loaded with Cal-520 (AAT Bioquest, California, U.S.A) by incubation with the acetoxymethyl (AM) ester form of the dye at a final concentration of 2 µM in culture media without serum. Dyes were prepared as 5 mM stock solution in DMSO and kept frozen at -20 °C until diluted. After 45 min, cells were washed several times with dye-free HEPES-buffered saline solution and transferred to an imaging chamber on a microscope (Zeiss Axio Examiner, Jena, Germany) equipped with a Zeiss 40x water immersion objective. Zen Blue imaging software (Carl Zeiss, Jena, Germany) was used for hardware control and image acquisition. Image analysis was performed using ImageJ (NIH, Maryland, U.S.A). All imaging experiments were performed at 34 °C in a low divalent cation-containing bath solution with the composition (in mM): 135 NaCl, 3 KCl, 0.5 CaCl_2_, 0.1 MgCl_2_, 10 HEPES and 10 glucose (pH 7.2; osmolality 290–300 mmol/kg). Images were acquired at 4 Hz. Background fluorescence was measured from the cell-free area outside the soma of interest in each frame of every time series. Region of interests were drawn around the soma and baseline fluorescence intensity (F0) was determined by averaging 24 frames preceding the cell’s exposure to BzATP or ATP and the time course of normalized fractional dye fluorescence [ΔF/F0] was obtained, where ΔF equals F(t) - F0.

### Western blot

hiPSC-derived astrocytes were homogenized in ice-cold extraction buffer (20 mM HEPES pH 7.4, 100 mM NaCl, 20 mM NaF, 1% Triton X-100, 1 mM sodium orthovanadate, 1 µM okadaic acid, 5 mM sodium pyrophosphate, 30 mM β-glycerophosphate, 5 mM EDTA, protease inhibitors (Complete, Roche, Cat. No 11,697,498,001)), electrophoresed on 8–10% sodium dodecyl sulphate (SDS)-polyacrylamide gel, transferred to nitrocellulose blotting membrane (Amersham Protran 0.45 μm, GE Healthcare Life Sciences) and blocked in TBS-T (150 mM NaCl, 20 mM Tris–HCl, pH 7.5, 0.1% Tween 20) supplemented with 5% non-fat dry milk. Membranes were incubated overnight at 4ºC with the rabbit anti-P2X7R antibody (Alomone Labs, Cat. Nr. APR-004, Jerusalem, Israel). On the next day, following washing with TBS-T, membranes were incubated with secondary HRP-conjugated IgG (Jackson Immuno Research, Plymouth, PA, U.S.A) and protein bands visualized using chemiluminescence. Gel bands were captured using a Fujifilm LAS-4000 imaging system (Fujifilm, Tokyo, Japan). GFAP (Merck, Missouri, U.S.A) was used as loading control. Hippocampal tissue from 8 week-old male P2X7R knock-out (KO) mice (C56BL/6 N-P2rx7^tm1d(EUKOMM)wtsi^) and corresponding wild-type (wt) mice (C67/Bl6 OlaHsd) was used as negative and positive control to test for P2X7R antibody specificity [16]. All animal experiments were performed in accordance with the principles of the European Communities Council Directive (2010/63/EU) and procedures were reviewed and approved by the Research Ethics Committee of the Royal College of Surgeons in Ireland (REC 1322) and Health Products Regulatory Authority (HPRA) (AE19127/P038).

### Statistical analysis

Data are expressed as mean ± SEM. Statistical analysis was performed using the Mann-Whitney U test. P < 0.05 was considered statistically significant. All statistical analyses was performed with GraphPad Prism 9 software (GraphPad Software, San Diego, CA, U.S.A).

## Results

### Expression of P2X7Rs in hiPSC-derived astrocytes

**Fig. 1:**
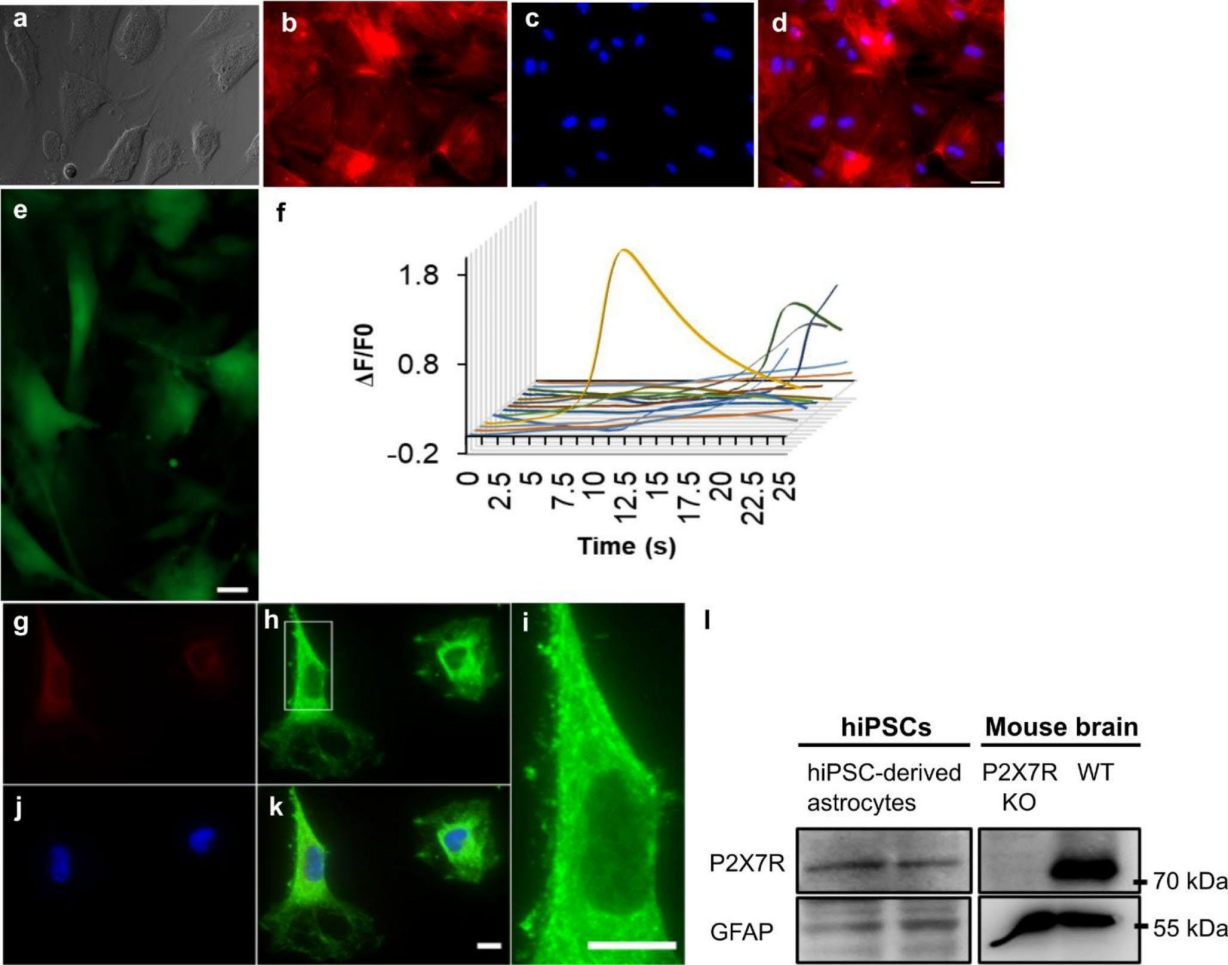
P2X7R expression in hiPSC-derived astrocytes. (**a**) Differentiated astrocytes. (**b**) Differentiated astrocytes express astrocyte marker S100β. (**c**) Nuclei stained with Hoechst (blue). (**d**) Merge of Hoechst (blue) and S100β (red). (**e**) Representative fluorescence image of astrocytes loaded with Cal-520. (**f**) Exemplary traces depicting spontaneous calcium transients in hiPSC-derived astrocytes. (**g**) Immunofluorescence analysis of hiPSC-derived astrocytes using antibodies directed against the P2X7Rs (**h,i,k**, green) with double-labelling using the astrocyte marker GFAP (**g**, red). Nucleus is counterstained with Hoechst (**j-k**, blue). (**l**) Western blot images of P2X7R protein expressed by hiPSC-derived astrocytes, and wild-type (WT) and P2X7R knock-out (KO) mouse hippocampi as positive and negative control. Scale bar 50 μm in **a-d** and **g-k** and **i**, and 20 μm in **e**

hiPSC-derived astrocytes, differentiated for 4 weeks in vitro and positive for the astrocyte marker S100β, showed a flat morphology (Fig. 1a-d). Astrocytes exhibited a weak signal for Cal-520 fluorescence indicating low resting [Ca^2+^]i with occasional spontaneous asynchronous [Ca^2+^]i fluctuations (Fig. 1f). Immunohistochemistry at 4 weeks in vitro showed a punctate staining pattern for P2X7R in GFAP-positive cells demonstrating the expression of P2X7Rs (Fig. 1g-i), which was further confirmed by Western blotting (Fig. 1l). Thus, astrocyte identity of the cells differentiated from pNSCs was validated by immunostaining for S100β which is a Ca^2+^-binding protein abundantly expressed in astrocytes, and GFAP which is a major inter-filamentous protein of mature astrocytes. Immunocytochemical analysis also revealed that 100% of Hoechst-positive nuclei were also GFAP-positive indicating a highly pure population of astrocytes.

### ATP evokes AFC-5128 or JNJ-47965567-sensitive Ca^*2+*^transients in iPSC-derived astrocytes

**Fig. 2:**
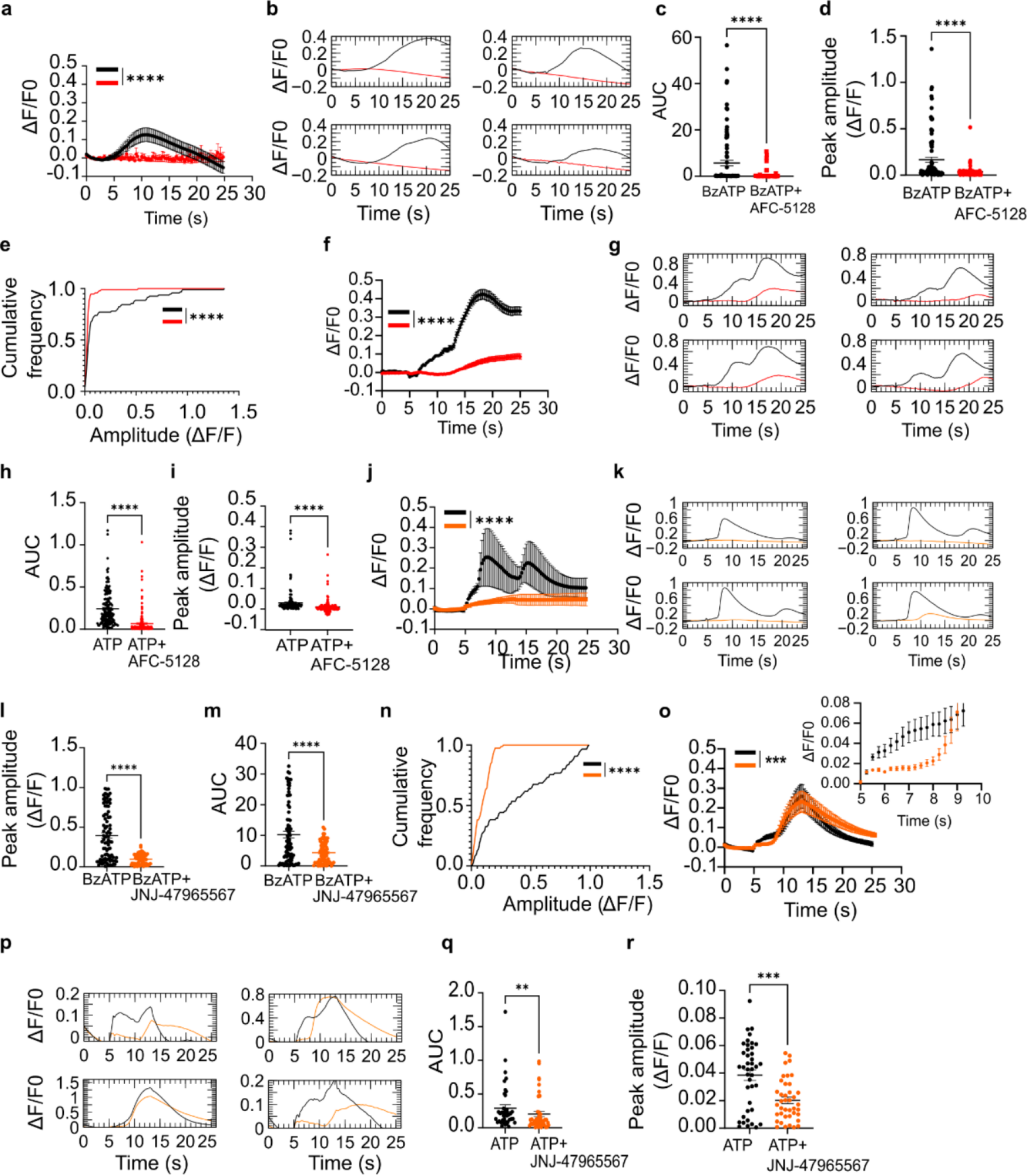
ATP-evoked Ca^2+^ responses in hiPSC-derived astrocytes. (**a**) Average time series showing response of astrocytes to the application of BzATP (300 µM) in the absence (black trace) and presence (red trace) of AFC-5128 (30 nM) (n = 96). (**b**) Exemplary ΔF/F0 traces illustrating the effect of AFC-5128 (30 nM) on BzATP-evoked calcium transients. (**c**) ATP-evoked Ca^2+^ area under the curve (AUC) during BzATP or BzATP and AFC-5128 application (n = 96). (**d**) Individual peak ΔF/F0 during BzATP or BzATP and AFC-5128 application (n = 96). (**e**) Cumulative frequency distribution of the amplitude of Ca^2+^ events during BzATP or BzATP and AFC-5128 application (n = 96). (**f**) Average time series showing response of astrocytes to the application of ATP (300 µM) in the absence (black trace) and presence (red trace) of AFC-5128 (30 nM) (n = 159). (**g**) Exemplary ΔF/F0 traces illustrating the effect of AFC-5128 (30 nM) on ATP-evoked calcium transients. (**h**) ATP-evoked Ca^2+^ AUC during ATP or ATP and AFC-5128 (n = 159). (**i**) Individual peak ΔF/F0 during ATP or ATP and AFC-5128 application (n = 159). (**j**) Average time series showing response of astrocytes to the application of BzATP (300 µM) in the absence (black trace) and presence (orange trace) of JNJ-47965597 (100 nM) (n = 85). (**k**) Exemplary ΔF/F0 traces illustrating the effect of JNJ-47965597 (100 nM) on BzATP-evoked calcium transients. (**l**) AUC of individual ΔF/F0 traces during BzATP or BzATP and JNJ-47965597 application (n = 85). (**m**) Individual peak ΔF/F0 during BzATP or BzATP and JNJ-47965597 application (n = 85). (**n**) Cumulative frequency distribution of the amplitude of Ca^2+^ events during BzATP or BzATP and JNJ-47965597 application (n = 85). (**o**) Average time series showing response of astrocytes to the application of ATP (300 µM) in the absence (black trace) and presence (orange trace) of JNJ-47965597 (100 nM) (n = 85). Inset shows magnified view of the Ca^2+^ response for the period of ATP application. (**p**) Exemplary ΔF/F0 traces illustrating the effect of JNJ-47965597 (100 nM) on ATP-evoked calcium transients. (**q**) ATP-evoked Ca^2+^ AUC during ATP or ATP and JNJ-47965597 application (n = 85). (**r**) Individual peak ΔF/F0 during ATP or ATP and JNJ-47965597 application (n = 85)). **p < 0.01; ***p < 0.001; ****p < 0.0001

Functional expression of P2X7Rs was assessed by monitoring changes in [Ca^2+^]i using the calcium-sensitive fluorescent reporter Cal-520. Pulse ejection of BzATP (300 µM) for 5 s evoked synchronous increase in [Ca^2+^]i persisting for a few seconds following agonist wash-out (Fig. 2a). In contrast, pre-incubation of astrocytes with the P2X7R antagonist AFC-5128 (30 nM), followed by co-application of BzATP with AFC-5128, reduced BzATP-mediated [Ca^2+^]i responses (Fig. 2a,b). The area under ΔF/F0 curve (AUC) upon BzATP application was reduced when stimulated in the presence of AFC-5128 (Fig. 2c). Similar results were also evident when the peak amplitude of individual ΔF/F0 traces were compared (Fig. 2d,e). Likewise, AFC-5128 also reduced ATP-induced [Ca^2+^]i rises, AUC of ΔF/F0 traces and peak amplitude (Fig. 2f-i). Similar to AFC-5128, the P2X7R antagonist JNJ-47965597 (100 nM) reduced BzATP and ATP-evoked [Ca^2+^]i signals (Fig. 2h, i), AUC and peak amplitude of individual ΔF/F0 traces (Fig. 2j-r). The averaged time course of BzATP or ATP-evoked [Ca]i shown in Fig. 2f, j and o shows a biphasic response probably caused by Ca^2+^-induced Ca^2+^ release. The attenuation of Ca^2+^ response in the presence of JNJ-47965597 for the period of ATP application is also shown in the magnified view in the inset (Fig. 2o). Since astrocytes were not mitotically inhibited, astrocytes at different maturation stages could account for the slightly different kinetics observed in response to the agonist application.

## Discussion

Here, we show that astrocytes respond by [Ca^2+^]i increase upon ATP and BzATP which was blocked via the selective P2X7R antagonists AFC-5128 or JNJ-47965567. Therefore, the present findings support the use of hiPSC-derived astrocytes as a human brain-relevant in vitro model for studying ATPergic signaling, disease mechanisms and drug screening.

To date, only two studies have reported the presence of *P2rx7* mRNA in cultured human adult and fetal brain tissue [14, 17]. Studies investigating a causative role of P2X7R in brain diseases have, however, relied heavily on experimental animal models. This raises potential problems with translation since the P2X7R exhibits remarkable species-specific differences in their pharmacological properties to drugs such as AFC-5128 [15]. hiPSC-derived glia have been widely accepted as human brain-relevant in vitro cellular models to investigate phenotypic alterations in neurological diseases [11, 18]. Thus, to advance findings from pre-clinical experimentation into the clinical setting, the development of human-derived cellular models for targeted drug screening, such as that for the P2X7R in this study, would serve an invaluable tool to bridge the gap between translational animal models and human neurological diseases.

The present study provides strong evidence that hiPSC-derived astrocytes express functional P2X7Rs. This is based on imaging evidence and the fact that P2X7R antagonists selectively blocked calcium entry. Our results therefore agree with studies showing functional P2X7R on astrocytes in mice, rats and humans [19], but are in contrast to other studies unable to detect P2X7R on rat and human astrocytes [5, 6] including work by our own group in mice [20].

It is important to keep in mind that our studies are carried out under in vitro conditions and whether P2X7Rs are expressed in vivo in human astrocytes requires further investigation (e.g. patch clamp in human brain slices). P2X7Rs have been shown to be upregulated in human reactive astrocytes [17]. We did not detect discernable changes in morphology of astrocytes in culture over time, but did not assess if the culture conditions lead to neuroinflammation and the generation of reactive astrocytes. BzATP-evoked responses should be interpreted with caution because BzATP is a non-specific agonist at purinergic receptors and agonist activity at non-P2X7Rs has been documented. Importantly, suggesting ATP- and BzATP-mediated responses are mainly mediated via P2X7Rs, the specific P2X7R antagonists AFC-5128 or JNJ-47965567 suppressed the observed calcium increases. Finally, our results should be studied in additional iPSC lines, from healthy individuals and patients carrying potential pathogenic gene variants.

In conclusion, here we demonstrate the expression of functional P2X7Rs on hiPSCs-derived astrocytes and provide the proof-of-concept that hiPSCs represent a valid model to study P2X7Rs signaling in human cellular models.

## Data Availability

The datasets used and/or analysed during the current study are available from the corresponding author on reasonable request.
